# Er:YAG laser irradiation surface effect of set bioceramic endodontic sealer – in vitro SEM study

**DOI:** 10.3389/fbioe.2025.1562578

**Published:** 2025-04-24

**Authors:** V. Stefanova, K. Zhekov

**Affiliations:** Department of Operative Dentistry and Endodontics, Faculty of Dental Medicine, Medical University of Plovdiv, Plovdiv, Bulgaria

**Keywords:** Er:YAG laser, bioceramic endodontic sealer, Bioroot RCS, laser irradiation, SEM

## Abstract

**Introduction:**

Recent developments in endodontics have led to increased interest in utilizing different methods for the removal of bioceramic endodontic sealers. The Er:YAG laser’s specific wavelength and energy delivery, we foresee its potential in laser-assisted removal of bioceramic sealers like BioRoot RCS.

**Aim:**

The primary aim is to examine the influence of Er:YAG laser irradiation on set BioRoot RCS and to discern and compare the effects on the sealer of two different time durations and two distinct laser settings.

**Materials and Methods:**

Specimens of BioRoot RCS (n = 20), each 10 mm in diameter and 3 mm thickness, were prepared according to manufacturer guidelines and set for 48 h at 37°C and 100% relative humidity. The “LiteTouch” Er:YAG laser system was applied with two different settings: 300 mJ, 5.10 W, 17 Hz, and water spray rate of 5, irradiation time 20 s for Group A (n = 5) and 40 s for Group B (n = 5). Settings of 250 mJ, 5.00 W, 20 Hz, with the same spray rate were used for 20 s irradiation for Group C (n = 5) and 40 s for Group D (n = 5). The laser sapphire tips employed were with dimensions D-1.3 mm and L-19 mm. Only half of each specimen underwent laser treatment, ensuring a controlled and comparative analysis of surface alterations and material removal, assessed using scanning electron microscopy on four different magnifications (x1,000; x2,000; x5,000 and x10,000).

**Results:**

All irradiated specimens demonstrated significant macroscopic and microscopic surface changes. We selected and compared scenograms of non-laser treated surfaces and laser treated surfaces of all experimental groups on the four magnifications. For Group A and Group B a greater effect on the material surface was observed in comparison with group C and Group D. Group B and Group D presented more substantial surface alterations compared to group A and Group C respectively.

**Conclusion:**

Er: YAG laser irradiation effectively alters the surface of the set BioRoot RCS. Longer irradiation time and bigger overall energy settings show superior changes. This pioneer *in vitro* investigation shows the possible use of lasers for bioceramic sealer removal in order to impact retreatment endodontic procedures.

## 1 Introduction

Recent developments in endodontics have led to increased interest in utilizing different methods and techniques for the removal of bioceramic endodontic sealers. Understanding the Er:YAG laser’s specific wavelength and energy delivery, we foresee its potential in laser-assisted removal of bioceramic sealers like BioRoot RCS. The influence of variables such as power, pulse duration, time for irradiation, and energy density is essential for the laser interaction with the material and the expected outcome.

In recent years, lasers have been widely utilized in dentistry for diverse applications, including caries detection, pulp vitality testing, and minimally invasive cavity preparation. Their ability to perform high-precision, low-damage procedures has extended to various surgical interventions and cosmetic treatments such as tooth bleaching and gingival contouring. Beyond clinical applications, lasers have garnered attention for their potential in modifying the surface properties of dental biomaterials. One critical factor in applying lasers within root canals is controlling temperature rise. Studies have shown that the Er:YAG laser, when used with appropriate energy settings and water spray cooling, can maintain safe temperature levels, avoiding damage to the periodontal ligament and surrounding bone. This makes it a safer alternative for intracanal procedures compared to other laser systems like Nd:YAG or CO_2_ lasers (Kuzekanani et al., 2019; Rossato et al., 2009; Ghoveizi et al., 2022; Stefanova et al., 2015; Meire and De Moor, 2024).

Dental biomaterials, designed to restore or replace damaged dental tissues, must maintain biocompatibility in the oral environment - a factor heavily influenced by surface characteristics such as roughness and wettability. By selectively adjusting laser parameters, it is possible to alter surface features at micro- and nanoscales, enhancing their interaction with biological tissues without compromising the material’s internal structure (Ghoveizi et al., 2022; Stefanova et al., 2015). Bioceramic materials, including sealers like BioRoot RCS, are increasingly favored in endodontics due to their excellent sealing properties, biocompatibility, and ability to bond to dentin. However, these advantages come with a significant drawback: once set, bioceramic sealers become highly solid and chemically stable, posing challenges during retreatment (Zhekov and Stefanova, 2021). Traditional methods for bioceramic sealer removal, such as rotary files and ultrasonic devices, often fail to fully eliminate the material (Zhekov and Stefanova, 2020). While ultrasonic devices have proven efficient in removing endodontic materials in general, lasers bring additional versatility to the field. Beyond their potential in bioceramic removal, lasers have been extensively used in modifying the surface of indirect ceramic restorations such as crowns, inlays, and onlays (Stefanova et al., 2015). They are also employed for etching enamel and dentin, activating irrigation solutions, and removing the smear layer from root canal walls (Kuzekanani et al., 2019; Anton et al., 2023). Despite these applications, there is limited clarity on whether direct laser exposure effectively destroys bioceramics. This knowledge gap underscores the importance of investigating the interactions between Er:YAG lasers and bioceramic sealers under controlled conditions.

The crystalline structure of the set BioRoot RCS bioceramic endodontic sealer primarily consists of hydration products resulting from its calcium silicate-based composition (Al-Haddad and Che Ab Aziz, 2016; Donnermeyer et al., 2019; Magnaudeix, 2022; El Hachem et al., 2024). Upon setting, the main crystalline phases include:

• Calcium Silicate Hydrate (C-S-H) Gel–This is the primary product formed during the hydration of tricalcium silicate (Ca_3_SiO_5_) and dicalcium silicate (Ca_2_SiO_4_). Although C-S-H is mostly amorphous or poorly crystalline, it provides the material with its mechanical strength and sealing ability.
• Calcium Hydroxide (Ca(OH)_2_) – Crystalline portlandite forms as a by-product of the hydration reaction. Its hexagonal crystal structure contributes to the material’s alkaline pH, enhancing its antimicrobial properties and promoting bioactivity through apatite formation.
• Hydroxyapatite (Ca_10_(PO_4_)_6_(OH)_2_) – Over time, the release of calcium ions and the interaction with phosphate ions in bodily fluids lead to the formation of hydroxyapatite crystals on the surface of the sealer. This hexagonal crystalline structure aids in biocompatibility and chemical bonding with dentin.
• Zirconium Oxide (ZrO_2_) – Present as a radiopacifier, zirconium oxide remains unchanged during the setting process. It has a monoclinic or tetragonal crystalline structure depending on its form and contributes to the sealer’s radiopacity without affecting its bioactivity.

The crystalline phases of set BioRoot™ RCS primarily include calcium hydroxide and gradually forming hydroxyapatite, while the C-S-H gel remains largely amorphous. The zirconium oxide remains crystalline and stable throughout.

The Er:YAG laser operates through a photothermal and photoacoustic mechanism. Its 2,940 nm wavelength is highly absorbed by water and hydroxyl groups in hard tissues, leading to micro-explosions that ablate the target material with minimal heat generation. This precise ablation minimizes thermal damage to surrounding tissues, which is critical in endodontic procedures where preservation of dentin integrity is essential (Gorduysus et al., 2017).

Bioceramic sealers like BioRoot™ RCS contain hydroxyl-rich phases such as calcium hydroxide and hydroxyapatite, making them potentially susceptible to Er:YAG laser energy. However, their high chemical stability presents a challenge. The Er:YAG laser’s optical effects may disrupt the crystalline matrix of these materials, facilitating their removal. This interaction requires investigation to determine the optimal laser settings (power, pulse duration, energy density) for effective ablation.

The need to investigate the use of Er:YAG lasers for bioceramic sealer removal is both timely and relevant. As the adoption of bioceramic materials continues to grow, so too does the demand for innovative techniques to overcome their inherent challenges. This study not only seeks to expand the understanding of laser-material interactions but also aims to contribute to the advancement of endodontic retreatment procedures, ultimately improving patient outcomes.

## 2 Aim

The primary aim is to examine the influence of Er:YAG laser irradiation on set bioceramic endodontic sealer BioRoot RCS. Specifically, the study aims to discern and compare the effects on the sealer when using two different time durations for laser irradiation and two distinct laser settings.

## 3 Materials and methods

### 3.1 Specimen preparation

Specimens of BioRoot RCS (n = 20) were prepared according to the manufacturer’s guidelines. One leveled scoop of the powder was mixed with five drops of the liquid on the smooth surface of a sterile glass pad with a sterile plastic spatula for approximately 60 s. Each specimen was prepared to a size of 10 mm in diameter and 3 mm thickness in a plastic mold to achieve uniformity. All specimen preparations were performed by the same operator [K.Z.] to prevent interoperator variations. After preparation, the specimens were set at 37°C and 100% relative humidity for 48 h to ensure full setting of the bioceramic material. This procedure mimics the typical clinical setting for the sealer’s curing process, allowing the material to harden before any laser treatment is applied.

### 3.2 Laser system and settings

The Er:YAG laser system “LiteTouch” was used for the laser treatments. This system was chosen due to its established efficacy in endodontic procedures and its ability to provide precise control over energy output. Based on the laser setting and time duration applied the specimens were divided into four experimental groups to assess the impact of varying laser parameters on the BioRoot RCS material.

• **Group A (n = 5)**: samples treated with 300 mJ, 5.10 W, 17 Hz, water spray rate of 5 and irradiation time 20 s.
• **Group B (n = 5)**: samples treated with 300 mJ, 5.10 W, 17 Hz, water spray rate of 5 and irradiation time 40 s.
• **Group C (n = 5)**: samples treated with 250 mJ, 5.00 W, 20 Hz, water spray rate of 5 and irradiation time 20 s.
• **Group D (n = 5)**: samples treated with 250 mJ, 5.00 W, 20 Hz, water spray rate of 5 and irradiation time 40 s.

For each of the experimental groups a new laser sapphire tip was used with a diameter D-1.3 mm and L-19 mm in length, selected for their optimal size to provide controlled and effective irradiation over the sample surface.

### 3.3 Experimental design

To ensure controlled and comparative analysis, only half of each specimen underwent laser treatment with the selected parameters, provided by a single operator [V.S.] to prevent interoperator variations. This design allows for a direct comparison between treated and untreated areas (as a control group) within the same specimen. By applying the laser to only one side of the sample, the impact of the laser on the material can be observed without interference from other treatment factors.

### 3.4 Scanning electron microscopy

Vacuum installations BH 30 were employed for carbon sputtering and thermal evaporation of metals, while the Mini Sputter Coater SC7620 was used for carbon evaporation, noble metal sputtering, and glow discharge. The scanning electron microscope (SEM) used for imaging was a Philips 515, which has been digitalized and is equipped with secondary electron image detectors (SEI). The SEM was operated at an acceleration voltage of 30 kV and can achieve a maximum magnification of ×40,000 for detailed imaging.

### 3.5 Surface alteration assessment

After laser irradiation, the treated and non-treated parts of each sample were observed for melting, ablation and cracks under scanning electron microscopy by two independent researchers and representative surface areas were recorded. A total of 160 scenograms were obtained under four different magnifications (×1,000, ×2,000, ×5,000, and ×10,000) and used to analyze the surface alterations. This approach provides a comprehensive view of the effects of laser irradiation, allowing for a comparison of both treated and non-treated areas, from general surface irregularities to fine details such as microfractures and material detachment.

### 3.6 Morphological analysis

The obtained SEM data were analyzed by comparing the morphological characteristics of non-treated BioRoot surfaces and laser irradiated ones. The morphological changes based on different laser settings and irradiation times were also assessed and compared.

## 4 Results

**FIGURE 1:**
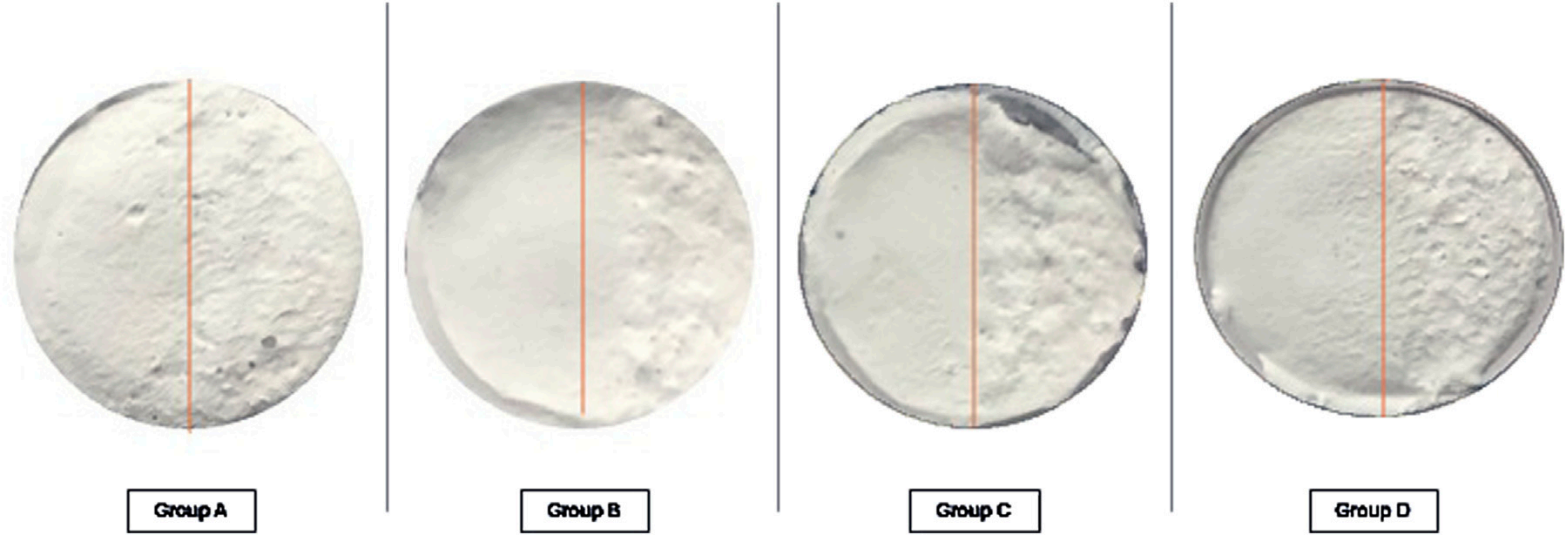
Macroscopic view of specimens by groups.

Non-irradiated halves of each of the tested samples exhibit smooth and even surface appearance. All irradiated specimens areas demonstrated significant macroscopic morphological changes (Figure 1).

**FIGURE 2:**
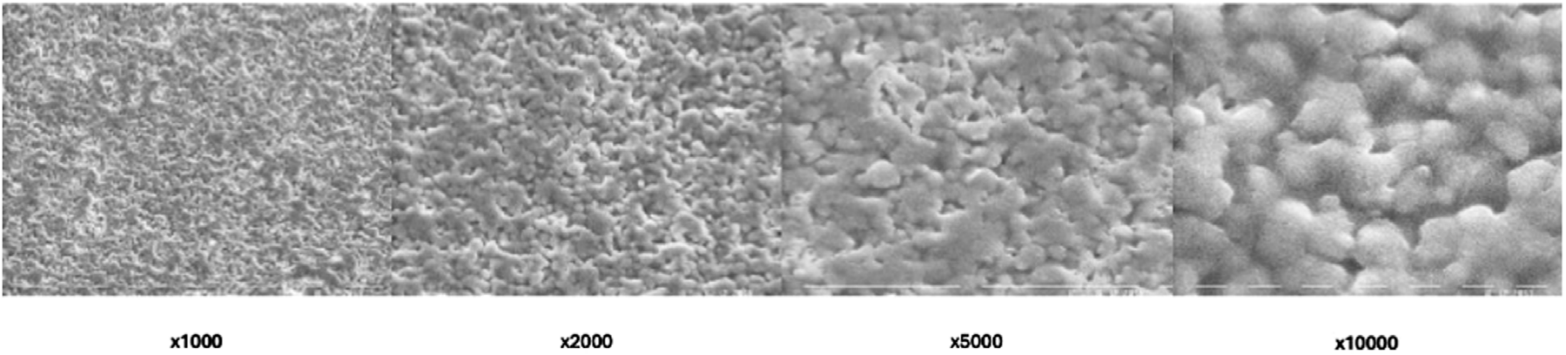
Scanning electron microscopic view of non-treated (control) BioRoot RCS surface under different magnifications.

SEM imaging was employed to assess the surface morphology of BioRoot RCS in its set non-treated form and provided detailed insights. The SEM analysis provided high-resolution images, allowing for a comprehensive evaluation of the material’s microstructure at a magnification of ×1,000, ×2,000, ×5,000 and ×10,000 (Figure 2).

The SEM images of the non-treated BioRoot RCS surface revealed a relatively smooth and homogenous structure, with no visible cracks or significant surface irregularities. The surface exhibited minimal porosity, suggesting that the material sets with a relatively compact and consistent structure. Fine microtopographical features were observed, indicating a microporous surface pattern that could potentially enhance biocompatibility and interaction with surrounding tissues. Some areas of the surface demonstrated slight textural variations, which might be attributed to the inherent characteristics of the material’s bioceramic composition.

**FIGURE 3:**
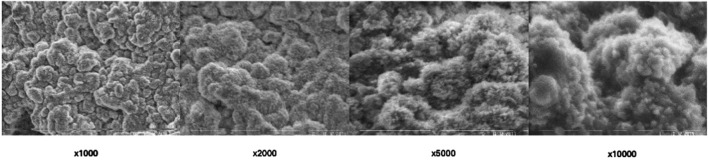
Scanning electron microscopic view of Group A under different magnifications.

**FIGURE 4:**
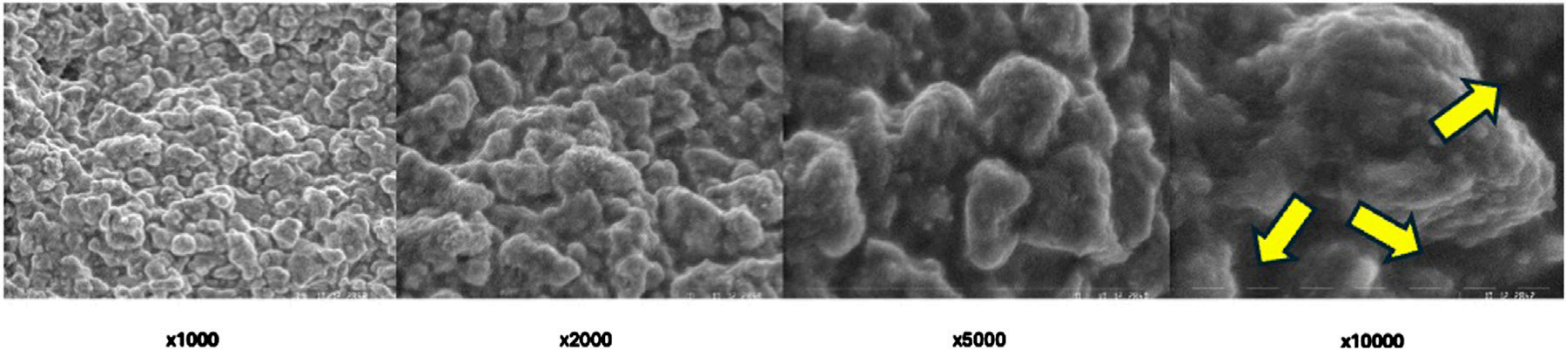
Scanning electron microscopic view of Group B under different magnifications.

**FIGURE 5:**
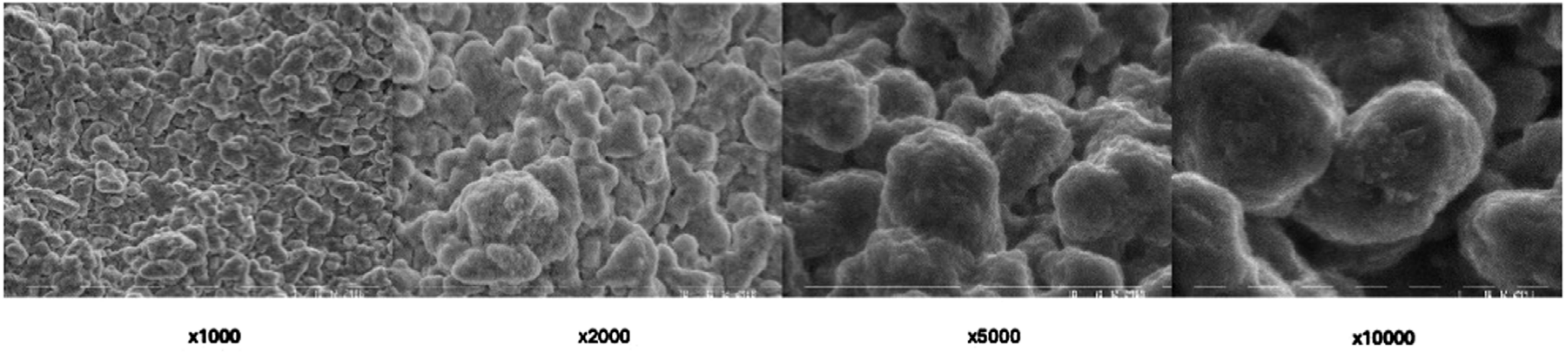
Scanning electron microscopic view of Group C under different magnifications.

**FIGURE 6:**
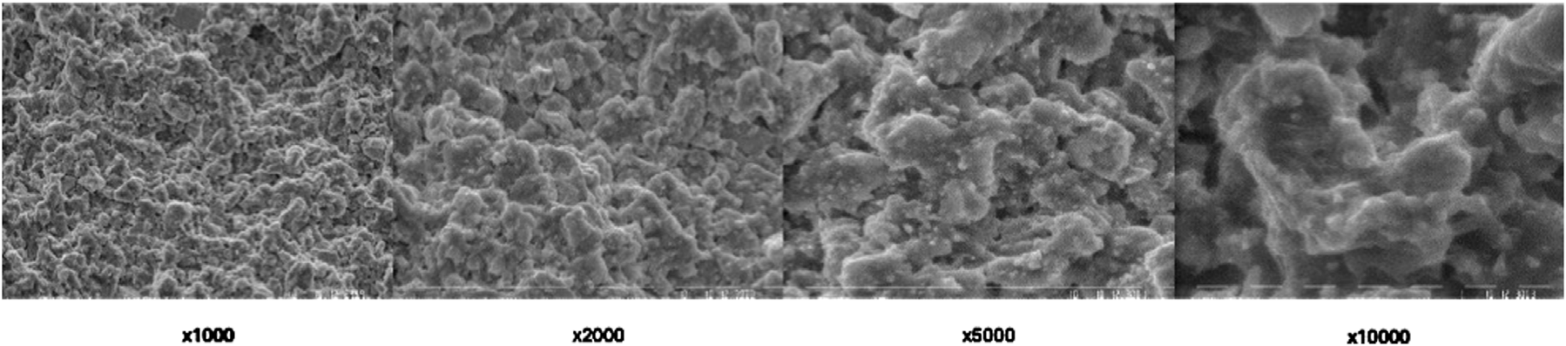
Scanning electron microscopic view of Group D under different magnifications.

SEM imaging provided detailed information about surface morphology alterations influenced by Er:YAG laser different settings (Figures 3–6). The SEM images offered high-resolution insights into the microstructural alterations induced by the laser irradiation, revealing distinct changes in the surface characteristics as a result of varying laser parameters, including irradiation time and laser intensity.

For samples irradiated with lower laser intensities and shorter exposure times (Group C), the SEM images revealed mild surface roughening, characterized by the development of subtle microvoids (Figure 5). The surface remained relatively intact, with minimal disruption of the underlying material structure. These alterations appeared to be localized and superficial, suggesting that lower intensity settings may lead to controlled, limited damage while preserving the integrity of the material.

In contrast, higher laser intensities and longer exposure times (Group A and Group D) resulted in more pronounced surface alterations. SEM analysis showed extensive surface roughening, with the formation of deeper microvoids (Figures 3, 6). The increased laser energy appeared to significantly disrupt the surface topography, leading to a more heterogeneous texture. These changes could potentially compromise the material’s structural integrity, as the surface exhibited signs of damage and material degradation.

At the highest intensity settings with prolonged irradiation times (Group B), the SEM images highlighted the extreme surface changes, including material erosion and the formation of larger voids visible on highest magnifications (Figure 4). On magnification ×10,000 arrows show the most eminent material loss. These extensive alterations indicated that excessive laser exposure might lead to undesirable structural damage, which could affect the material’s mechanical properties.

Overall, SEM imaging provided valuable qualitative data regarding the impact of different Er:YAG laser settings on the surface morphology of set BioRoot RCS. The results indicate that while moderate laser parameters may induce slight roughening, more intense or prolonged laser irradiation can lead to severe surface degradation, which may hinder the material’s performance in clinical applications.

In any area of interest in the treated parts of each sample surface alterations were evident. No cracks and/or melting were found.

### 4.1 Energy settings comparison

Groups A and B (300 mJ, 5.10 W) demonstrated greater surface alterations compared to Groups C and D (250 mJ, 5.00 W). The higher energy setting effectively enhanced material disruption and ablation.

### 4.2 Irradiation time comparison

Longer irradiation times (40 s in Groups B and D) produced more substantial surface changes than shorter times (20 s in Groups A and C). This suggests a cumulative effect of energy application over time.

### 4.3 Microscopic observations

SEM analysis revealed distinct morphological changes across groups. Lower magnifications (×1,000, ×2,000) highlighted general surface irregularities, while higher magnifications (×5,000, ×10,000) exposed increased porosity, and material loss.

## 5 Discussion

### 5.1 Mechanisms of interaction

The study highlights the potential of Er:YAG laser irradiation in altering the surface morphology of bioceramic sealer–BioRoot RCS. The interaction mechanism between Er:YAG laser and BioRoot RCS is driven by its 2,940 nm wavelength, which corresponds to the absorption peaks of water and hydroxyapatite (Parker, 2007). Due to the hydrophilic nature of BioRoot RCS and its moisture content, the Er:YAG laser energy is efficiently absorbed, leading to localized heating, vaporization, and material ablation. This mechanism is supported by the presence of water within the bioceramic matrix, which facilitates efficient energy transfer and enhances the laser’s ablative effect (Marciano et al., 2016). Since BioRoot RCS is highly hydrophilic the explosive vaporization mechanism ensures effective removal with minimal residual heat effects. Er:YAG lasers have been previously utilized for dentin etching and final irrigation procedures in endodontics, where the smear layer is removed from root canal walls (Meire and De Moor, 2024; Anton et al., 2023). The laser’s ability to interact with water-rich structures is instrumental in achieving these outcomes. The findings of this study extend these principles to bioceramic sealers, demonstrating their potential for material removal in retreatment scenarios.

### 5.2 Influence of laser parameters

Higher energy settings (300 mJ, 5.10 W) and prolonged exposure (40 s) induced more significant surface alterations compared to lower energy settings (250 mJ, 5.00 W), consistent with previous literature (Gorduysus et al., 2017; Saran et al., 2023). Specifically, the 300 mJ, 5.10 W setting employed in Groups A and B generated greater ablative effects compared to the 250 mJ, 5.00 W setting used in Groups C and D. Doubling the irradiation time from 20 to 40 s amplified these effects, underscoring the importance of exposure duration in achieving desired outcomes. Scanning electron microscopy provided critical insights into the morphological changes induced by laser treatment. SEM analysis revealed dose-dependent surface alterations. At ×1,000–×2,000, general disruptions and voids were visible, while at ×5,000–×10,000, microstructural damage such as microcracks and porosity was evident. These microstructural changes may affect the mechanical integrity of the sealer, while the bonding properties to dentine might be a topic for another investigation.

### 5.3 Comparison with literature

Previous studies have examined the efficacy of various laser systems on dental materials in both restorative dentistry and endodontics. While Er:YAG has been extensively studied on composite resins and zirconia, its application to bioceramic sealers remains largely unexplored. Rossato et al. (2009) demonstrated that Er:YAG laser treatments on aged composite resin restorations produce outcomes comparable to traditional methods such as diamond burs and sandblasting (Rossato et al., 2009). Similarly, Ghoveizi (2022) investigated the effect of different power levels of the Er:YAG laser on the bond strength of zirconia to resin cement, again comparing these results to sandblasting (Ghoveizi et al., 2022). Stefanova et al. (2015) explored Er:YAG laser functionalization for composite indirect restorations, further reinforcing the versatility of this technology in restorative applications (Stefanova et al., 2015). Our findings extend previous research to a novel material category relevant to endodontic retreatment.

Building on these foundations, our study introduces a novel application of the Er:YAG laser to bioceramic materials, specifically BioRoot RCS. Unlike earlier research that primarily focused on composites, zirconia, or restorative materials, this work shifts the focus to bioceramic sealers and their laser-induced surface alterations in the context of endodontic retreatment. This marks a significant step forward, as it represents the first investigation into the laser’s effects on such materials, providing new insights into its potential in endodontic procedures.

The findings from our study align with prior work, confirming the utility of Er:YAG lasers in endodontic applications (Meire and De Moor, 2024; Anton et al., 2023; Gorduysus et al., 2017; Kapetanović et al., 2022). However, our research stands apart by addressing a different class of materials and their unique challenges. This distinction is further underscored by Garibb and Camilleri’s recent contributions (Garrib and Camilleri, 2020). Their research expanded on the known difficulties of removing bioceramic sealers, highlighting their chemical stability and resistance to conventional retreatment methods. By identifying these limitations, they emphasized the need for alternative removal techniques. Our study builds on these insights, showing that laser irradiation may offer a solution to these challenges. Preliminary observations indicate that Er:YAG laser treatment could effectively overcome the mechanical and chemical barriers identified by Camilleri and Garibb (Gorduysus et al., 2017; Kapetanović et al., 2022). The high reflectivity of ZrO_2_ in the infrared spectrum reduces laser absorption, likely contributing to the uneven ablation patterns observed. This limitation should be considered in clinical application (Sari et al., 2014). Nonetheless, further quantitative investigations and comparative *in-vitro* and *in-vivo* studies remain necessary to fully establish the laser’s efficacy and compare it against other removal methods, such as ultrasonic or rotary instrumentation.

### 5.4 Clinical implications

Nowadays dentists use bioceramic endodontic sealers routinely (Stefanova et al., 2023). The Er:YAG laser demonstrated potential as an adjunctive tool for the removal of bioceramic sealers during endodontic retreatment, a procedure currently challenged by the material’s high adhesion and resistance to mechanical methods. The ability of the Er:YAG laser to effectively alter the surface morphology of bioceramic sealers like BioRoot RCS positions it as a promising tool for retreatment procedures. Bioceramic sealers are known for their strong adhesion to dentinal walls and resistance to conventional removal techniques, posing challenges during retreatment (Zhekov and Stefanova, 2024). The laser’s ability to disrupt and ablate these materials offers a potential solution, facilitating more efficient and effective removal.

Additionally, the precision and control afforded by laser systems could minimize damage to surrounding dentinal tissues, preserving tooth structure and improving treatment outcomes. However, the technique’s clinical adoption will require further validation through *in vivo* studies, as well as the development of standardized Er:YAG laser protocols in clinical settings to ensure safety and efficacy. This may lead to potential for tooth structure preservation due to laser selectivity.

## 6 Conclusion

This study underscores the importance of optimizing laser parameters, including energy density and exposure duration, to achieve desired outcomes. Within the limitations of this study we may conclude that:

1. Er:YAG laser irradiation effectively alters the surface morphology of set BioRoot RCS.
2. Higher energy settings and longer irradiation times produce more pronounced changes in the material.
3. Er:YAG lasers show promise for bioceramic sealer removal, underscoring their versatility in endodontic procedures.

Further research is needed to evaluate the technique’s efficacy *in vivo*, compare its performance to other removal methods, and develop standardized clinical protocols.

## Data Availability

The raw data supporting the conclusion of this article will be made available by the authors, without undue reservation.
